# A systematic review of radiological outcomes and implant positioning in robotic-assisted functionally aligned robotic total knee arthroplasty

**DOI:** 10.1051/sicotj/2025068

**Published:** 2026-01-28

**Authors:** Vasileios Giovanoulis, Angelo V. Vasiliadis, Eleftherios Tsiridis, Luca Andriollo, Pietro Gregori, Konstantinos Dretakis, Christos Koutserimpas, Sébastien Lustig

**Affiliations:** 1 Orthopaedics Surgery and Sports Medicine Department, Hôpital de la Croix-Rousse Lyon France; 2 Third Academic Department of Orthopaedics, Papageorgiou General Hospital Thessaloniki 56403 Greece; 3 Orthopedic Surgery Department, Lyon Ortho Clinic, Clinique de la Sauvegarde Lyon France; 4 Ortopedia e Traumatologia, Fondazione Poliambulanza Istituto Ospedaliero Via Bissolati 57 25124 Brescia Italy; 5 Fondazione Policlinico Universitario Campus Bio-Medico Roma Italy; 6 Second Department of Orthopaedics, Hygeia General Hospital of Athens Greece; 7 School of Health Rehabilitation Sciences, University of Patras Greece; 8 IFSTTAR, LBMC UMR_T9406, Univ Lyon, Claude Bernard Lyon 1 University, Lyon France

**Keywords:** Total knee arthroplasty, Functional positioning, Functional alignment, Robotic-assisted TKA, Component positioning

## Abstract

*Introduction*: Functional alignment (FA) or functional knee positioning is a patient-specific strategy for total knee arthroplasty (TKA) that utilizes robotics to balance coronal, sagittal, and axial planes while preserving joint-line orientation and soft-tissue tension within predefined guardrails. Although early clinical outcomes are encouraging, the radiographic profile and workflow consistency of robotic FA have not been clearly synthesized. *Methods*: In accordance with PRISMA guidelines, English-language studies of primary robotic FA-TKA with ≥2-year follow-up were searched. Eligible designs included RCTs, prospective/retrospective cohorts, and large case series (≥50 patients). Information on pre- and postoperative coronal alignment [hip–knee–ankle angle (HKA), lateral distal femoral angle (LDFA), medial proximal tibial angle (MPTA)], component positioning (femoral valgus/rotation/flexion; tibial varus/rotation/slope), and explicit FA workflow boundaries (guardrails) was extracted. *Results*: Twenty-one cohorts (5,360 knees) reported at least one radiographic or workflow endpoint. Preoperatively, the predominant deformity was varus. Postoperatively, limb alignment converged near neutral: HKA clustered around 178–179.5°, with LDFA ~89–91° and MPTA ~87–89°. Component positions were tightly distributed within FA targets: femoral valgus ≈ 0.5–1.5°, tibial varus ≈ ~3°, femoral flexion ~6–9°, and tibial slope ~0–3°; tibial rotation was overwhelmingly referenced to Akagi’s line, and femoral rotation to the TEA in most series. Reported guardrails showed strong convergence: typical workflows included femoral valgus −3° to +6°, tibial varus 0–6°, tibial slope 0–3°, and femoral ER ~3–6° to TEA. Across cohorts, achieved radiographs closely tracked these limits, indicating high adherence and reproducibility. Most observational studies had a moderate risk of bias; the lone RCT was low risk. *Discussion*: Robotic FA-TKA delivers a radiographic profile with slight femoral valgus and modest tibial varus, while keeping components within narrow, pre-specified guardrails. *Level of evidence*: Level III, systematic review and meta-analysis.

## Introduction

Functional alignment (FA), also described as functional knee positioning, has emerged as a patient-specific alignment philosophy for total knee arthroplasty (TKA) that seeks to reproduce native joint lines and soft-tissue tension in three dimensions rather than forcing a uniform mechanical neutral axis [1, 2]. FA tailors component orientation to each patient’s native limb morphology and ligamentous behavior, aiming to recreate physiological joint-line orientation and balanced gaps across coronal, sagittal, and axial planes [3]. The concept emphasizes joint-line preservation and kinematic harmony with the least possible soft-tissue release [4].

This level of individualization is greatly facilitated by robotic assistance [5]. Image-based planning and intraoperative analytics allow surgeons to iteratively adjust bone resections and component positions while observing gap symmetry in extension and flexion in real time [3, 6]. In practice, FA is implemented within predefined “guardrails” (workflow boundaries) that cap coronal alignment, tibial slope, and axial rotation (referenced to established axes), thereby enabling personalization without drifting into extreme positions [2, 3].

Despite encouraging clinical outcomes, the radiographic profile delivered by FA remains incompletely defined. Interpretation is limited by heterogeneous measurement conventions (rotational and slope references, neutral definitions, imaging modality, and intra-op vs post-op reporting) and by subgroup-only reporting instead of whole-cohort aggregates [7–9]. These inconsistencies make meta-analytic pooling difficult and highlight the need for a structured synthesis describing typical postoperative limb alignment (hip–knee–ankle angle [HKA], lateral distal femoral angle [LDFA], medial proximal tibial angle [MPTA]) and component positioning (femoral valgus/rotation/flexion; tibial varus/rotation/slope).

Accordingly, the objective of this systematic review was to consolidate radiological outcomes and intraoperative workflow parameters in robotic FA-TKA. The authors aimed to summarize (1) pre- and postoperative limb alignment (HKA, LDFA, MPTA), (2) femoral and tibial component positioning, including the rotational references used, and (3) the alignment boundaries that characterize FA workflow.

## Materials and methods

This systematic review was conducted in accordance with the Preferred Reporting Items for Systematic Reviews and Meta-Analyses (PRISMA) guidelines [10]. The protocol was prospectively registered with PROSPERO (CRD420251134340). A comprehensive search of PubMed/MEDLINE and Scopus was performed from database inception to September 2025 using the following string: (“total knee arthroplasty” OR “TKA” OR “total knee replacement” OR “TKR”) AND (“functional alignment” OR “functional knee position” OR FA OR FKP) AND (robotic OR “robotic-assisted” OR “robot-assisted”). The search was restricted to English-language publications. Reference lists of all eligible full-text articles were manually screened to identify additional studies.

Studies were eligible if they enrolled adult patients undergoing primary robotic-assisted TKA performed under FA principles and reported outcomes at a minimum follow-up of two years. Acceptable designs were randomized controlled trials, prospective or retrospective comparative cohort studies, and large case series with at least 50 patients. To ensure clinical relevance, studies were required to report at least one validated clinical outcome and, for the purpose of the present analysis, to include radiological alignment and/or component-positioning data or explicit descriptions of the FA workflow. Exclusion criteria were revision or partial knee arthroplasty, small case series with fewer than 50 patients, cadaveric/biomechanical investigations, expert opinions, narrative reviews, and non-English publications.

Study selection was performed independently by two reviewers (V.G., A.V.V.) who screened titles and abstracts and then assessed full texts; disagreements were resolved by discussion with a third reviewer (C.K.). Data extraction was completed independently by the same reviewers using a standardized form. Only the information on the FA groups (from comparative studies with other alignment philosophies) was recorded.

The radiological variables of interest were pre- and postoperative coronal alignment, including the hip–knee–ankle angle (HKA), the lateral distal femoral angle (LDFA), and the medial proximal tibial angle (MPTA) and component positioning outputs and limits: femoral valgus, femoral external-rotation reference, femoral flexion, tibial varus/valgus, tibial external-rotation reference, and tibial posterior slope. Workflow fields comprised the boundaries used in the robot for each plane (for example, 0–6° tibial varus, Akagi’s [11] line for tibial rotation, 0–3° tibial slope, and degrees for femoral external rotation), together with any stated alignment philosophy or guardrails. Outcomes were synthesized descriptively due to heterogeneous frames of reference (e.g., transepicondylar axis (TEA) vs posterior condylar axis (PCA); Akagi’s line vs soft-tissue referencing).

Continuous variables were extracted as means with standard deviations when available; medians with interquartile ranges or ranges were recorded verbatim. Because radiographic angles and rotational references were reported heterogeneously across studies (using different axes and tolerances), outcomes were synthesized primarily through descriptive statistics (typical values, ranges, and frequencies of targets/guardrails) rather than being pooled quantitatively. When units or reference frames differed, values were summarized within their native reference system without transformation to avoid misclassification. Categorical data were presented as counts and percentages.

Risk of bias was assessed at the study level using the RoB 2 tool for randomized controlled trials and the ROBINS-I tool for non-randomized studies. Overall certainty of the evidence was judged as very low, low, moderate, or high considering risk of bias, inconsistency, indirectness, imprecision, and potential publication bias.

## Results

**Figure 1:**
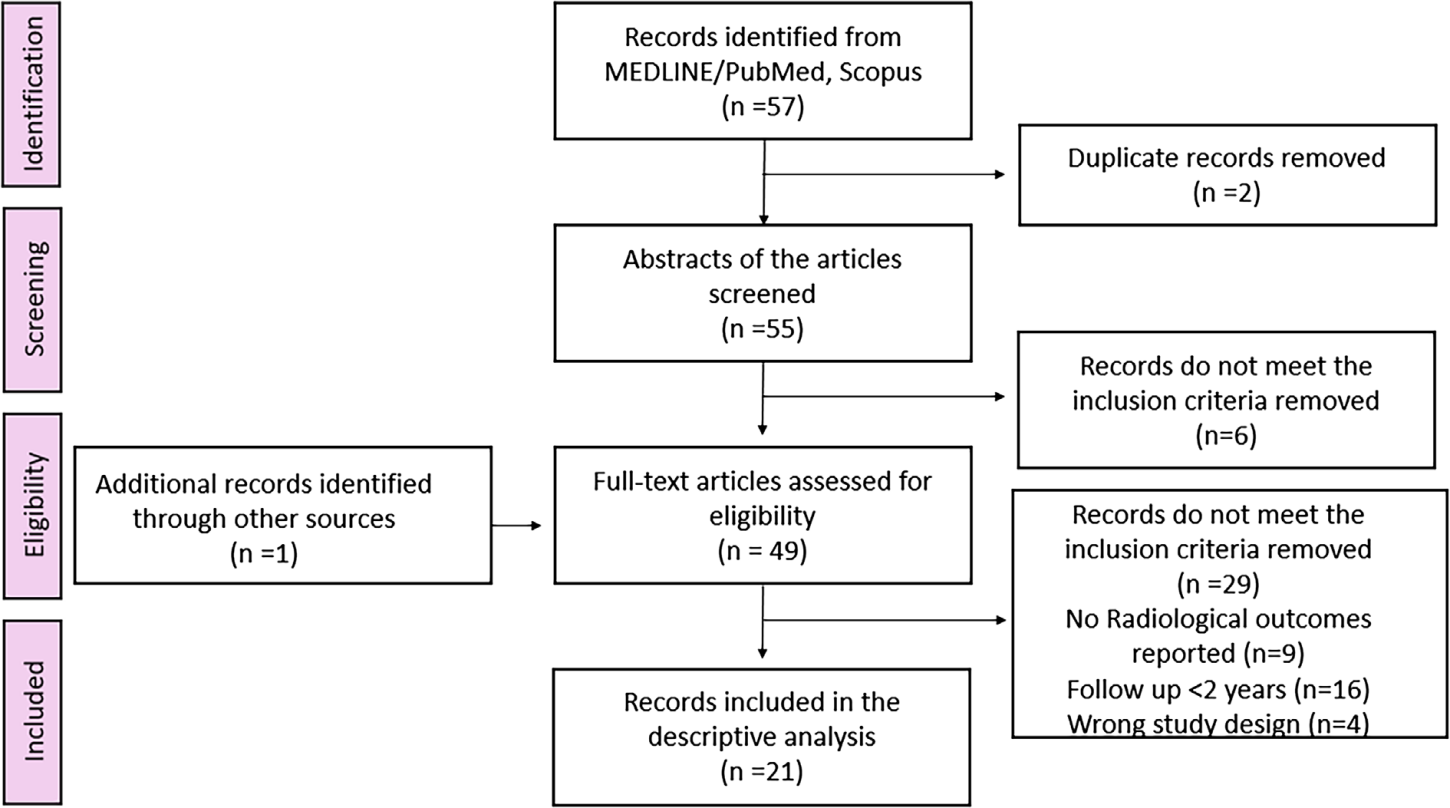
Preferred Reporting Items for Systematic Reviews and Meta-analyses (PRISMA) flowchart.

All 21 FA–robotic TKA cohorts (5360 knees) [7, 8, 12–30] in the radiology dataset reported at least one alignment or component-positioning endpoint (Figure 1). Mean age across the cohorts clustered around 66–72 years, Body Mass Index (BMI) typically ~26–32 kg/m², and women comprised roughly ~50–66% of participants; all series used an image-based MAKO platform.

Preoperatively, coronal deformity was predominantly varus: studies that provided HKA typically showed means around 170–176° with LDFA ~88–91° and MPTA ~85–87° where available. Postoperatively, limb alignment converged slightly varus, close to neutral, across cohorts. Where HKA was reported, most means/medians lay around 178–179.5°. LDFA generally centered at ~89–91° and MPTA at ~87–89° (Table 1).

**Table 1:** Pre- and post-operative radiological alignment data among the included studies.

Study	Pre-operative			Post-operative		
	HKA	LDFA	MPTA	HKA	LDFA	MPTA
Andriollo et al. [12]*	174.1 ± 5.5	89.3 ± 5.8	86.3 ± 3.1	ΔTS 0–5°: 177.9 ± 2.8	ΔTS 0–5°: 89.4 ± 2.2	ΔTS 0–5°: 88 ± 2.3
				ΔTS >5°: 177.9 ± 3	ΔTS >5°: 89.3 ± 2.5	ΔTS >5°: 87.9 ± 2.3
Andriollo et al. [13]*	174.1 ± 5.5	89.3 ± 5.8	86.3 ± 3.1	CF ≤7.5°: 177.6 ± 2.9	CF ≤7.5°: 89.7 ± 2.4	CF ≤7.5°: 88.2 ± 2.1
				CF >7.5°: 178.1 ± 3	CF >7.5°: 89 ± 2.3	CF >7.5°: 87.9 ± 2.5
Andriollo et al. [14]*	173.2 ± 3.9	89.1 ± 5.8	86 ± 2.8	NR	NR	NR
Choi et al. [15]*	170.7 ± 5.7	90.5 ± 2.8	85.4 ± 3.2	178.9	96.3° ± 2.1	89.2° ± 1
Clark et al. [7]*	FAm: 179.1	FAm: 87.2	FAm: 86.3	FAm: 178.7	FAm: 89.7	FAm: 88.4
	FAk: 179	FAk: 86.9	FAk: 85.9	FAk: 178.5	FAk: 87.5	FAk: 86
Daffara et al. [16]	NR	NR	NR	NR	NR	NR
Diquattro et al. [17]	NR	NR	NR	NR	NR	NR
Diquattro et al. [18]	NR	NR	NR	NR	NR	NR
Koutserimpas et al. [19]*	F: 175	F: 91	F: 87	F: 179	F: 91	F: 88
	M: 174	M: 91	M: 86	M: 178	M: 90	M: 88.1
Koutserimpas et al. [20]^#^	Varus: 173	Varus: 91	Varus: 86	Varus: 178	Varus: 91	Varus: 88
	Valgus: 186	Valgus: 93	Valgus: 90	Valgus: 181	Valgus: 90	Valgus: 89
Koutserimpas et al. [21]^#^	≤3°: 176	≤3°: 91	≤3°: 87	≤3°: 179	≤3°: 91	≤3°: 89
	>3°: 172	>3°: 91	>3°: 85	>3°: 177	>3°: 91	>3°: 87
Koutserimpas et al. [22]^#^	BMI ≥30: 175	BMI ≥30: 91	BMI ≥30: 87	BMI ≥30: 178	BMI ≥30: 91	BMI ≥30: 88
	BMI <30: 175	BMI <30: 91	BMI <30: 87	BMI <30: 179	BMI <30: 90.5	BMI <30: 89
Koutserimpas et al. [23]^#^	CS: 174	CS: 91	CS: 87	CS: 179	CS: 91	CS: 88
	PS: 176	PS: 91	PS: 87	PS: 179	PS: 91	PS: 88
Koutserimpas et al. [24]^#^	CL: 175	CL: 91	CL: 87	CL: 179	CL: 91	CL: 88
	CM: 177	CM: 91	CM: 88	CM: 179	CM: 90	CM: 89
Koutserimpas et al. [25]^#^	≥10°: 174	NR	NR	≥10°: 178	NR	NR
	<10°: 178			<10°: 178		
Koutserimpas et al.[26]^#^	NR	NR	NR	rFKP: 175	NR	NR
				unFKP: 174		
Manara et al. [27]	NR	NR	NR	NR	NR	NR
Nixon et al. [8]*	<2 mm: 178.6 ± 2.6	<2 mm: 86.9 ± 1.9	<2 mm: 86.5 ± 2	NR	NR	NR
	2–3 mm: 177.8 ± 2.4	2–3 mm: 87.5 ± 1.8	2–3 mm: 86.2 ± 1.9			
	3–6 mm: 177.9 ± 2.4	3–6 mm: 87.4 ± 1.9	3–6 mm: 85.9 ± 1.9			
Yang et al. [28]*	170.5 ± 5.4	88.6 ± 3	85.1 ± 3.3	NR	NR	NR
Young et al. [29]*	FA: 175.5 ± 6.23	FA: 87.7 ± 2	FA: 87 ± 2.7	FA: 179.5 ± 2.73	FA: 87.4 ± 2.1	FA: 87.2 ± 1.7
	MA: 175.7 ± 5.75	MA: 87.9 ± 2.3	MA: 87.1 ± 2.4	MA: 179 ± 2.38	MA: 89.6 ± 1.5	MA: 90.9 ± 0.9
Yu et al. [30]*	171.3 ± 4.7	NR	NR	NR	NR	NR

Data are expressed as mean* or median^#^ (degrees of angle) ± SD.

Abbreviations: HKA, hip-knee-ankle; LDFA, lateral distal femoral angle; MPTA, medial proximal tibial angle; ΔTS, Δ tibial slope; CF, combined flexion; FAk, functional alignment kinematic; FAm, functional alignment mechanical; F, female; M, male; BMI, body mass index; CS, cruciate-substituting; PS, posterior stabilize; CL, cementless; CM, cemented; rFKP, restricted functional knee positioning; unFKP, unrestricted functional knee positioning; FA functional alignment; MA, mechanical alignment; NR, not reported.

**Figure 2:**
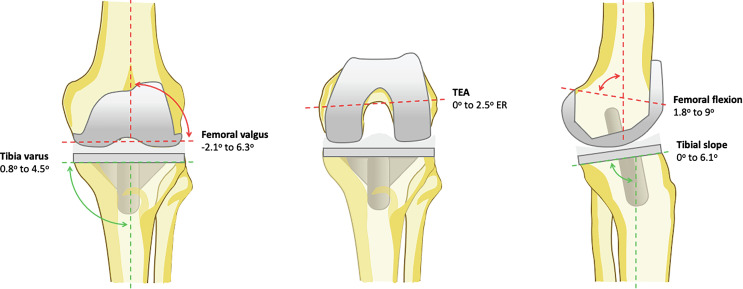
The mean values of the 3-D implant positioning from the reviewed studies.

Component positioning outputs were narrowly distributed and aligned with FA goals [3] which targets HKA 174°–180°, femoral positioning from 3° varus to 6° valgus, tibial positioning at 0°–6° varus, femoral rotation 0° internal rotation–6° external rotation. Femoral valgus clustered around ~0.5–1.5°, femoral external rotation values were small (often ~0–0.5° when a single mean was given), and femoral flexion was typically ~6–9° (e.g., Andriollo et al. 6.6–6.9° [12–14]; multiple Koutserimpas et al. strata 6.2–9° [12, 20–25]). Tibial coronal placement concentrated around ~3° varus and was stable across subgroups such as age, BMI, or patellar strategy [20, 22], while the posterior tibial slope was usually ~0.7–1.0°. Tibial external rotation was referenced almost uniformly to Akagi’s line, sometimes expressed simply as “Akagi’s line” and other times with an explicit narrow tolerance band (Figure 2, Table 2).

**Table 2:** Femoral and tibial component positioning data among the included studies.

	Femoral component			Tibial component		
Study	Valgus	ER	Flexion	Varus	ER	Posterior slope
Andriollo et al. [12] *	0.5° ± 1.8°	0.2° ± 1.85°	6.9° ± 2.6°	3.3° ± 1.7°	NR	0.8° ± 0.8°
Andriollo et al. [13] *	0.5° ± 1.8°	0.2° ± 1.85°	6.9° ± 2.6°	3.3° ± 1.7°	NR	0.8° ± 0.8°
Andriollo et al. [14] *	0.5° ± 2°	0.2° ± 1.7°	6.6° ± 2.7°	3.4° ± 1.7°	NR	0.7° ± 0.8°
Choi et al. [15] *	6.3°	2°	NR	0.8°	NR	2.6°
Clark et al. [7]*	NR	NR	NR	NR	NR	NR
Daffara et al. [16]	NR	NR	NR	NR	NR	NR
Diquattro et al. [17]	NR	NR	NR	NR	NR	NR
Diquattro et al. [18]	NR	NR	NR	NR	NR	NR
Koutserimpas et al. [19]*	F: 1.1°	F: 0	F: 8.1°	F: 3°	NR	F: 0
	M: 0.3°	M: 0.3°	M: 6°	M: 3.5°		M: 1°
Koutserimpas et al. [20]^#^	Varus: 0.7°	Varus: 0.1°	Varus: 7°	Varus: 3.5°	Varus: 0	Varus: 1°
	Valgus: 1.5°	Valgus: 0.5°	Valgus: 9°	Valgus: 1°	Valgus: 0	Valgus: 0
Koutserimpas et al. [21]^#^	≤3°: 0.7°	≤3°: 0	≤3°: 7.05°	≤3°: 2°	≤3°: 0	≤3°: 1°
	>3°: 0.7°	>3°: 0.3°	>3°: 7°	>3°: 4.5°	>3°: 0	>3°: 1°
Koutserimpas et al. [22]^#^	BMI ≥30: 0.9°	BMI ≥30: 0	BMI ≥30: 7.25°	BMI ≥30: 3°	BMI ≥30: 0	BMI ≥30: 1°
	BMI <30: 0.9°	BMI <30: 0.35°	BMI <30: 7.7°	BMI <30: 3.5°	BMI <30: 0	BMI <30: 1°
Koutserimpas et al. [23]^#^	CS: 0.9°	CS: 0	CS: 7.9°	CS: 3.5°	CS: 0	CS: 1°
	PS: 1°	PS: 0.1°	PS: 7.8°	PS: 3°	PS: 0	PS: 0
Koutserimpas et al. [24]^#^	CL: 1°	CL: 0	CL: 7.5°	CL: 3.5°	CL: 0CM: 0	CL: 1°CM: 0.5°
	CM: 0.95°	CM: 0.3°	CM: 7.55°	CM: 2.25°		
Koutserimpas et al. [25]^#^	≥10°: 0	≥10°: 0	≥10°: 6.2°	≥10°: 4°	NR	≥10°: 1°
	<10°: 1°	<10°: 0.2°	<10°: 7°	<10°: 3.5°		<10°: 0
Koutserimpas et al.[26]^#^	rFKP: −2.1°	rFKP: 1.8°	rFKP: 4.4°	rFKP: 1°	NR	rFKP: 1°
	unFKP: −1.1°	unFKP: 0.4°	unFKP: 7°	unFKP: 4.5°		unFKP: 1°
Manara et al. [27]	NR	NR	NR	NR	NR	NR
Nixon et al. [8]*	NR	6°	NR	NR	NR	NR
Yang et al. [28]*	0.9° ± 1.9°	2.5° ± 2.4°	1.8° ± 1.8°	4.2° ± 1.6°	NR	6.1° ± 1.1°
Young et al. [29]*	FA: NR	FA: NR	NR	FA: NR	NR	FA: NR
	MA: 0	MA: 0		MA: 0		MA: 3°
Yu et al. [30]*	NR	NR	NR	NR	NR	NR

Data are expressed as mean* or median^#^ (degrees of angle) ± SD.

Abbreviations: ER, external rotation; IR, internal rotation; F, female; M, male; BMI, body mass index; CS, cruciate-substituting; PS, posterior stabilize; CL, cementless; CM, cemented; rFKP, restricted functional knee positioning; unFKP, unrestricted functional knee positioning; FA functional alignment; MA, mechanical alignment; NR, not reported.

Workflow guardrails showed striking convergence across studies that stated them explicitly. Typical femoral ranges were valgus 6° to varus 3° [7, 8, 19–22, 24, 25, 27–30], external/internal rotation (ER/IR) ± 6/3° [12–14, 16–22, 24, 25, 27, 28] from transepicondylar axis (TEA), and flexion 0–10° across all works except one study that ranged 0–7° [7].Tibial rotation was overwhelmingly referenced to Akagi’s line [7, 8, 12–14, 17–24, 25, 29], with a few series [15, 28, 30] describing soft-tissue balancing as the operative reference; tibial ranges commonly included 0–6° varus except from Daffara [16] ranged between 4° varus and 2° valgus; Choi [15] positioned from 3° varus to 3° valgus; Yang [28] kept between 5° varus and 5° valgus and Koutserimpas [26] applied two tibial-varus strategies: 0–3° (Group A) and 0–6° (Group B). Posterior slope ranged in all studies between 0 and 3° (Table 3).

**Table 3:** The boundaries for tibia and femoral implant during total knee arthroplasty among included studies.

Study	Tibia varus	ref-TR	Tibia slope	Femoral valgus	Femoral flexion	Femoral int/rot	ref-FR
	0 to 6°	Akagi’s line	0 to 3°	0° to LDFA°	0 to 10°	3° to 6°	TEA
Andriollo et al. [12]*	0 to 6°	Akagi’s line	0 to 3°	0° to LDFA°	0 to 10°	3° to 6°	TEA
Andriollo et al. [13]*	0 to 6°	Akagi’s line	0 to 3°	0° to LDFA°	0 to 10°	3° to 6°	TEA
Andriollo et al. [14]*	0 to 6°	MA: s/t balancing	0 to 7°	±3°	NR	NR	MA: s/t balancing
		FA: NR					FA: TEA
Choi et al. [15]*	3° varus to 3° valgus	Akagi line	0 to 7°	−3° to 6°	0 to 7°	−6° to 6°	TEA
Clark et al. [7]*	0 to 6°	NR	0 to 3°	0 to 6°	0 to 5°	3° to 6°	TEA
Daffara et al. [16]	4° varus to 2° valgus	Akagi’s line	0 to 3°	0 to 6°	0 to 10°	3° to 6°	TEA
Diquattro et al. [17]	0 to 6°	Akagi’s line	0 to 3°	0 to 6°	0 to 10°	3° to 6°	TEA
Diquattro et al. [18]	0 to 6°	Akagi’s line	0 to 3°	0° to LDFA°	0 to 10°	3° to 6°	TEA
Koutserimpas et al. [19]*	0 to 6°	Akagi’s line	0 to 3°	−3° to 6°	0 to 10°	3° to 6°	TEA
Koutserimpas et al. [20]^#^	0 to 6°	Akagi’s line	0 to 3°	−3° to 6°	0 to 10°	3° to 6°	TEA
Koutserimpas et al. [21]^#^	0 to 6°	Akagi’s line	0 to 3°	−3° to 6°	0 to 10°	3° to 6°	TEA
Koutserimpas et al. [22]^#^	0 to 6°	Akagi’s line	0 to 3°	−3° to 6°	0 to 10°	3° to 6°	TEA
Koutserimpas et al. [23]^#^	0 to 6°	Akagi’s line	0 to 3°	−3° to 6°	0 to 10°	3° to 6°	TEA
Koutserimpas et al. [24]^#^	0 to 6°	Akagi’s line	0 to 3°	−3° to 6°	0 to 10°	3° to 6°	TEA
Koutserimpas et al. [25]^#^	0 to 6°	NR	0 to 3°	NR	NR	NR	TEA
Koutserimpas et al.[26]^#^	Groupe A: 0 to 3°	NR	0 to 3°	−3° to 6°	0 to 10°	3° to 6°	NR
	Groupe B: 0 to 6°						
Manara et al. [27]	0 to 6°	Akagi’s line	0 to 3°	−3° to 6°	0 to 10°	±6°	PCA
Nixon et al. [8]*	0 to 6°	s/t balancing	0 to 3°	−3° to 6°	0 to 10°	3° to 6°	TEA
Yang et al. [28]*	5° varus to 5° valgus	Akagi’s line	0 to 7°	−3° to 6°	0 to 10°	−6° to 3°	TEA
Young et al. [29]*	0 to 6°	s/t balancing	0 to 3°	−3° to 6°	0 to 10°	±3°	TEA
Yu et al. [30]*	0 to 6°	Akagi’s line	0 to 3°	−3° to 6°	0 to 10°	3° to 6°	TEA

Data are expressed as mean* or median^#^ (degrees of angle) ± SD.

Abbreviations: ref-TR, reference for tibial rotation; int/rot, internal rotation; ref-FR, reference for femoral rotation; NR, not reported; s/t balancing, soft-tissue balancing; LDFA, lateral distal femoral angle; MA, mechanical alignment; FA functional alignment; TEA, surgical trans-epicondylar axis; PCA, posterior condylar axis.

### Study quality (ROBINS-I)

Risk-of-bias assessments for the included observational cohorts were primarily moderate (19 cohorts), with a small number of studies rated as low-moderate (2 cohorts). The sole randomized controlled trial (RCT) was assessed as having a low risk of bias based on the ROB2 tool used for randomized studies

## Discussion

The principal finding of this review is that robotic FA in TKA in predominantly varus knees achieves slightly varus- near to neutral reproducible radiographic targets. Across more than 5,000 FA knees with a minimum of 2 years’ follow-up, limb alignment clustered near neutral (HKA ≈178–179.5°) with component placement concentrated in narrow bands (femoral valgus ≈0.5–1.5°, tibial varus ≈3°, femoral flexion ≈6–9°, tibial slope ≈0–3°). These achieved positions show that although FA is a personalized alignment strategy using the soft tissue envelope of the knee as guidance, it leads to safe radiographic targets [31].

Mechanical alignment (MA) targets a straight mechanical axis, classically HKA 180° ± 3°, with femoral and tibial components implanted perpendicular to their respective mechanical axes and has the virtues of standardization and durability data [32, 33]. However, imposing neutral on every knee can disregard native joint-line obliquity and soft-tissue laxities, sometimes necessitating collateral releases and risking non-physiologic kinematics in anatomies that deviate from neutral [34]. In contrast, FA individualizes the femoral and tibial cuts, according to the mediolateral laxities in extension and flexion, within explicit limits (e.g., tibial varus 0–6°, femoral valgus −3° to +6°, tibial slope 0–3°, femoral ER ~3–6° to TEA), aiming to preserve the patient’s phenotype while keeping components inside safety bounds [3]. The radiological outcomes synthesized in this systematic review show that FA usually lands slightly “constitutional”, with subtle femoral valgus and modest tibial varus; yet the overall limb remains near-neutral, aligning with contemporary survivorship goals.

Kinematic Alignment (KA) seeks to “resurface the knee” and fully restore the native joint lines with minimal or no releases by preserving native femoral anatomy [35]. Adjustments, if needed, are preferentially made on the tibial side [36]. While KA may yield favorable kinematics and patellofemoral tracking, valgus morphotypes may be driven toward excess femoral valgus and internal rotation, risking trochlear under-coverage or malorientation unless implants or limits are adapted [37, 38]. However, FA leverages image-based robotic analytics to restore joint-line obliquity and to restore the anterior compartment of the TKA [39–42]. Notably, the FA cohorts in this review achieved almost neutral-leaning HKA and constrained component positions without signals of instability or anterior-compartment overstuffing [42] at early follow-up [9].

These concerns have motivated “restricted KA” (rKA), which retains the kinematic intent but introduces caps to avoid extremes. As described by Vendittoli [43], rKA applies the kinematic philosophy but imposes explicit limits to avoid extreme positions. In practice, rKA caps coronal deviation to ≤5° at both the femur and tibia and keeps the overall limb at HKA ~180° ± 3°. The goal is to preserve the native (often oblique) joint line and respect each patient’s anatomy while preferentially adapting corrections on the tibial side when fine-tuning is needed. Building on rKA, FA keeps the respect for native joint-line obliquity but operationalizes it with robot-defined guardrails and real-time gap analytics, shifting from “restore with caps” to “personalize” in three dimensions and positioning within limits [44].

Thus, FA represents a three-dimensional conceptual approach to TKA in which planning and execution are guided by bony morphology, extension–flexion gap behavior, and patellofemoral kinematics. The trochlea is deliberately oriented while the ligaments are preserved, using controlled adjustments in femoral rotation and sagittal positioning to approximate the patient’s native trochlear geometry, thereby promoting balanced gaps and physiologic soft-tissue tension [45, 46]. Given that FA is a personalized alignment strategy, it is expected to preserve the patient’s underlying knee morphotype. In the studies included, the preoperative coronal profile was predominantly varus (typical HKA 170–176°, LDFA 88–91°, MPTA 85–87°). Under FA, these parameters were adjusted toward neutral but not fully neutralized, resulting in constitutionally oriented postoperative radiographs (HKA ~178–179.5°, LDFA ~89–91°, MPTA ~87–89°). This pattern reflects the intended preservation of each knee’s native morphotype while achieving balanced alignment. Component positions mostly stayed inside narrow guardrails (femoral valgus ~1°, tibial varus ~3°, posterior slope ~0–1°, tibial rotation referenced to Akagi’s line).

This review has several important limitations. First, most included cohorts were retrospective, single-center series, and only one randomized trial was available; these features raise the possibility of selection and reporting bias. Most cohorts were single-center, retrospective, with relatively short follow-up; thus, the relationship between these radiographic targets and longer-term wear or loosening cannot be fully assessed. Cohorts mixed means with medians/IQRs without raw data, precluding robust pooling. The timing of postoperative imaging was inconsistent, and few studies reported inter-/intra-observer reliability or blinded measurements. Finally, potential overlap between institutional cohorts and the predominance of high-volume centers may temper generalizability.

In conclusion, across 21 cohorts, robotic FA consistently produced a radiographic profile that was adjusted toward neutrality while still reflecting each patient’s native morphotype. Component positions clustered tightly within the predefined guardrails, indicating high adherence and reproducibility of the FA workflow. Collectively, these findings suggest that FA, when executed with image-based robotic analytics, reliably restores joint-line orientation and balanced alignment without overcorrecting towards absolute neutrality or drifting into malalignment.

## Data Availability

Data is available upon reasonable request to the corresponding author.
